# Is there a causal effect of parity on body composition: a birth cohort study

**DOI:** 10.1186/s12889-018-5089-2

**Published:** 2018-02-22

**Authors:** Bárbara Reis-Santos, Fernando C. Barros, Bernardo L. Horta

**Affiliations:** 1 Postgraduate Programme in Epidemiology, Federal University of Pelotas, Marechal Deodoro, 1160, 3° piso, Centro, Pelotas, Rio Grande do Sul 96020-220 - Caixa Postal 464 Brazil; 2 Laboratory of Epidemiology, Federal University of Espírito Santo, Marechal Campos S/N, Santos Dumont, Vitória, Espírito Santo, 29040-091 Brazil; 3 Postgraduate Program in Health and Behavior, Catholic University of Pelotas, Gonçalves Chaves, 373, Centro, Pelotas, Rio Grande do Sul 96015-560 Brazil

**Keywords:** women health, reproductive factors, obesity, body mass index, waist circumference, fat mass

## Abstract

**Background:**

Non-communicable diseases are the leading cause of death, worldwide. Obesity is one of the factors that is associated with the development of such diseases. The role of reproductive factors on women body composition has been evaluated, but the findings are controversial. This study was aimed at assessing the association of parity with body composition among women.

**Methods:**

In 1982, the maternity hospital of Pelotas, a southern Brazilian city, were visited daily and all deliveries were identified. Those livebirths whose family lived in the urban area of the city have been prospectively followed (*n* = 5914). In 2012–13, we tried to follow the whole cohort, the subjects were interviewed and examined. We evaluated the association of parity with the following body composition variables: body mass index, waist circumference and fat mass %. Estimates were adjusted for family income, skin color, maternal schooling, occupational status, alcohol, smoking, physical activity, and consumption of processed and ultraprocessed foods. All these analyses were replicated among the cohort men as a comparison. We also assessed whether duration of breastfeeding moderated the association.

**Results:**

In the 2012–13 visit, 3701 subjects were evaluated (mean age of 30.2 years). In the present analysis, we included 1620 women and 1653 men. 33% of women were nulliparous and 48% of men were without children. Even after controlling for confounding, parous women had a BMI 0.96 kg/m^2^ (95% CI: 0.30; 1.62) higher than nulliparous and for men the regression coefficient was 0.79 kg/m^2^ (95% CI: 0.29; 1.29). Waist circumference was also higher among parous women. Among men, the association was not linear and the regression coefficients were lower than that observed among women [3.41 cm (95% CI: -0.91; 7.73) among men and 4.83 cm (95% CI: 2.43; 7.24) among women with more than 3 children when compared with those without children], but this difference was not statistically significant (interaction *p* value = 0.58). Fat mass % was not associated with parity. Breastfeeding did not modify the association between parity and body composition.

**Conclusions:**

Parity was positively associated with body mass index and waist circumference among women. However, similar results among men suggest that there is no causal effect of parity.

**Electronic supplementary material:**

The online version of this article (10.1186/s12889-018-5089-2) contains supplementary material, which is available to authorized users.

## Background

Non-communicable diseases are the leading cause of death, worldwide [1]. Obesity is one of the factors that is associated with an increased risk of developing such diseases. The population attributable fraction for all-cause mortality due to overweight or obesity ranges from 5% in east Asia to 19% in North America [1]. Because the prevalence of overweight is rising [2], these estimates tend to increase in the next years. For this reason, it is relevant to identify obesity risk factors.

The role of reproductive factors on women body composition has been evaluated, but the results are controversial. It has been hypothesized that biological changes due to pregnancy would lead to a later unbalance on women body composition [3]. A recently published meta-analysis reported a higher odds of obesity among women with high parity [4]. However, since there was high heterogeneity among the studies and most of them had no adequate adjustment for confounders, the authors were not able to establish whether the association was causal or a consequence of residual confounding by sociodemographic, environmental, and/or behavioral variables [4].

Determining causation is a challenge that has always been shaped by the limitation of available data and the understanding of the underlying process [5]. In this sense, the assessment of the association between parity and body composition among men would be a strategy to improve causal inference [6, 7]. Disparities in the effect of parity among women and men would suggest that physiologic mechanisms within the female reproductive system are involved in the association between parity and body composition [8]. Conversely, similar results among men and women would suggest that this association is likely due to lifestyle factors or residual confounding [8]. To our knowledge, only one study [6] used this strategy. Hardy et al. (2007) reported that body mass index (BMI) and waist to hip ratio were higher among those women who had four or more children and the regression coefficients were higher among women when compared with men. Because the confidence intervals included the reference, the observed associations could be due to random [6]. Therefore, further studies using such strategy should be carried out.

This study was aimed at assessing the association of parity with body composition among women who have been prospectively followed since birth, in a southern Brazilian city. This association was also evaluated among men to increase causal inference.

## Methods

In 1982, the maternity hospitals of Pelotas, a southern Brazilian city, were visited daily and all deliveries were identified. Those liveborns (5914) whose families lived in the urban area were examined and their mothers were interviewed [9]. These subjects have been prospectively followed [10, 11].

In 2012, we tried to follow the whole cohort and the subjects were invited to visit the research clinic [11]. In this visit, an interviewer gathered information on sociodemographic, behavioral, reproductive, and health related variables. Anthropometric assessment was also carried out and the participants were asked to donate a blood sample. With respect to parity, the cohort members were asked about the number of lives births.

The anthropometric evaluation was carried out by previously trained and standardized assessors. Weight was measured using a calibrated scale with a precision of 100 g and height with a portable stadiometer with a precision of 0.5 cm. BMI (kg/m^2^) was calculated by dividing the weight in kilograms by height in square meters. Waist circumference was measured halfway between the lowest costal edge and the ipsilateral iliac crest. These measures were assessed twice (acceptable error lower than 1 cm between the measures) and the average of these measures was used in the analyses. When the error was higher than the acceptable, a third measurement was performed. Fat mass (%) was evaluated using dual-energy x-ray absorptiometry (DXA).

Socioeconomic status was evaluated by family income, maternal schooling, and skin color. Total income, in Brazilian reais, earned by family members in the last month was recorded and posteriorly categorized into monthly minimum wages (Brazil’s monthly minimum wage in 2012–13 was equivalent to $308). Maternal schooling in complete years of schooling was collected in the perinatal study, skin color and occupational status was self-reported by the cohort members.

Concerning behavioral characteristics, we evaluated current alcohol consumption; tobacco smoking (those subjects who smoked at least one cigarette for week, were considered as smokers); physical activity [evaluated using the long version of the International Physical Activity Questionnaire (IPAQ) and those subjects who reported more than 150 min /week of walking or physical activity of moderate-vigorous intensity (occupational and leisure-time domains) were considered as active]. The daily consumption of processed and ultra-processed foods (calories) was estimated from the interviewer-applied and computerized food frequency questionnaire [12]. The latter evaluated the participants’ annual intake of 88 food items [12]. We considered processed foods any food that has been altered from its natural state in some way, either for safety reasons or convenience [13, 14]. Ultra-processed food result from the processing of several foodstuffs, including ingredients from processed and unprocessed or minimally processed basic foods [13, 14]. Information on the number of months that the women breastfed each child was also obtained in the 2012 visit.

We used Chi-square test to compare proportions and analysis of variance to evaluate differences between means. Linear regression models were used to assess the association between parity and body composition. Adjusted models were determined a priori, according to a theoretical model based on previous literature. They included the covariates family income, skin color, maternal schooling, occupational status, alcohol, smoking, physical activity, and consumption of processed and ultra-processed foods. In the regression models, we assessed the normality of residuals and homoscedasticity. Also, collinearity between independent variables was evaluated using the variance inflation factor. All these analyses were replicated among the cohort men.

Furthermore, we also assessed whether duration of breastfeeding moderated the association between parity and body composition. Data was analyzed using Stata 14 (Stata Corp., College Station, USA). The study was approved by the ethics committee of the Universidade Federal de Pelotas and all participants signed an informed consent form.

## Results

In the 2012–13 visit, 3701 subjects were evaluated (mean age of 30.2 years). Added to the 325 deaths identified among the cohort members, represented a follow-up rate of 68.1%. In the present analysis, we included 1620 women and 1653 men assessed in 2012–13 (428 subjects were excluded from the analysis because information on the outcomes was missing or inconsistent). Table 1 shows that 33% of women were nulliparous and 48% of men were without children. With respect to income, 46% of women and 36% of men had a family income of three or less minimum wages. 23% of women and 6% of men were unemployed. Concerning smoking, 39% and 43% of women and men, respectively, had ever smoked.

**Table 1:** Characteristics of the studied sample according to gender

Characteristics	Women n (%)	Men n (%)
Parity		
0	534 (33)	799 (48)
1	494 (30)	508 (31)
2	319 (20)	248 (15)
3	149 (9)	69 (4)
≥ 4	124 (8)	29 (2)
Maternal schooling – years		
0–4	514 (32)	522 (32)
5–8	701 (43)	731 (44)
9–11	176 (11)	183 (11)
≥ 12	229 (14)	217 (13)
Family income – minimum wages		
0–1	145 (9)	65 (4)
1.001–3	594 (37)	533 (32)
3.001–5	375 (23)	472 (29)
> 5	506 (31)	583 (35)
Skin color		
White	1257 (78)	1236 (75)
Black	244 (15)	266 (16)
Brown	71 (4)	94 (6)
Yellow/Indigenous	48 (3)	57 (3)
Occupational status		
Unemployed	376 (23)	103 (6)
Employed	1244 (77)	1550 (94)
Alcohol consumption		
No	754 (47)	434 (26)
Yes	866 (53)	1219 (74)
Smoking		
No	984 (61)	947 (57)
Yes	636 (39)	706 (43)
Physically active^a^		
No	799 (49)	670 (41)
Yes	821 (51)	983 (59)
Daily ultraprocessed and processed foods consumption – cal, mean (sd)	796 (580)	884 (755)
Mean body mass index (kg/m^2^)	26.7 (5.9)	27.0 (5.0)
Mean waist circumference (cm)	80.5 (11.8)	89.3 (11.7)
Mean fat mass %	39.3 (8.4)	24.2 (8.7)
Total, n	1620	1653

*sd* standard deviation

^a^Subjects who reported more than 150 min /week of walking and physical activity of moderate-vigorous intensity (occupational and leisure-time domains)

Table 2 shows the association of parity with confounding variables. Maternal schooling and family income were inversely associated with parity among women. The prevalence of alcohol consumption and subjects who were physically active (overall physical activity, including occupational and leisure time) were higher among nulliparous women. Among men, maternal schooling and household income were also higher among those without children. The proportion of employed men was positively associated with parity (*p* < 0.001). Male subjects who had ever smoked were more prevalent among those with more than three children (*p* = 0.001).

**Table 2:** Parity according to socioeconomic and behavioral variables

Characteristics	Women – Parity					*p* value
	0 (*n* = 534)	1 (*n* = 494)	2 (*n* = 319)	3 (*n* = 149)	≥4 (*n* = 124)	
Maternal schooling – years						
0–4	20	32	41	44	44	< 0.001
5–8	38	48	43	46	43	
9–11	14	11	9	5	11	
≥ 12	28	9	7	5	2	
Family income – minimum wages						
0–1	4	7	13	13	22	< 0.001
1.001–3	23	38	42	52	55	
3.001–5	23	25	26	20	14	
> 5	50	30	19	15	9	
Skin color						
White	84	77	74	70	71	< 0.001
Black	11	17	18	19	16	
Brown	3	4	4	5	10	
Yellow/Indigenous	2	2	4	6	3	
Employed	84	79	71	69	61	< 0.001
Alcohol consumers	60	52	48	50	48	0.004
Ever smoked	28	39	44	55	57	< 0.001
Physically active	56	47	48	48	51	0.026
Daily ultraprocessed and processed foods consumption – cal, mean (sd)	699 (545)	785 (478)	881 (685)	878 (626)	948 (673)	< 0.001
	Men – Parity					
	0 (*n* = 799)	1 (*n* = 508)	2 (*n* = 248)	3 (*n* = 69)	≥4 (*n* = 29)	
Maternal schooling – years						
0–4	25	33	42	54	45	< 0.001
5–8	43	47	43	38	48	
9–11	13	10	10	1	7	
≥ 12	19	10	5	7	0	
Family income – minimum wages						
0–1	4	3	5	4	3	< 0.001
1.001–3	27	34	39	49	45	
3.001–5	26	31	30	28	45	
> 5	43	32	26	19	7	
Skin color						
White	78	73	73	64	69	0.063
Black	14	17	19	17	14	
Brown	4	6	6	13	14	
Yellow/Indigenous	3	4	2	6	3	
Employed	91	96	96	99	100	< 0.001
Alcohol consumption	74	74	75	70	69	0.873
Ever smoked	38	45	47	58	55	0.001
Physically active	62	57	56	67	59	0.203
Daily ultraprocessed and processed foods consumption – cal, mean (sd)	793 (592)	936 (845)	1024 (889)	989 (1099)	1044 (574)	< 0.001

*sd* standard deviation

Parity was positively associated with body mass index, among women and men, and the regression coefficients were similar. Even after controlling for confounding, parous women had a BMI 0.96 kg/m^2^ (95% confidence interval: 0.30; 1.62) higher than nulliparous and, among men with children the regression coefficient was 0.79 kg/m^2^ (95% confidence interval: 0.29; 1.29) higher than among those without children. Waist circumference was also higher among parous women. Among men, the association was not linear and the regression coefficients were lower than that observed among women [3.41 cm (95% confidence interval: − 0.91; 7.73) among men and 4.83 cm (95% confidence interval: 2.43; 7.24) among women with more than 3 children when compared with those without children]. However, this difference was not statistically significant (interaction *p* value = 0.58). Fat mass % was not associated with parity (Table 3).

**Table 3:** Crude and adjusted regression coefficients of the association of parity and body composition outcomes

	Body mass index (kg/m^2^)				Waist circumference (cm)				Fat mass (%)			
Parity	Women		Men		Women		Men		Women		Men	
	Unadjusted	Adjusted^a^	Unadjusted	Adjusted^a^	Unadjusted	Adjusted^a^	Unadjusted	Adjusted^a^	Unadjusted	Adjusted ^a^	Unadjusted	Adjusted^a^
Parity												
0	Ref.	Ref.	Ref.	Ref.	Ref.	Ref.	Ref.	Ref.	Ref.	Ref.	Ref.	Ref.
1	1.01 (0.29–1.73)	0.68 (−0.06–1.43)	0.55 (− 0.01–1.10)	0.77 (0.21–1.33)	2.56 (1.12–3.99)	1.79 (0.30–3.28)	0.79 (− 0.51–2.10)	1.35 (0.04–2.66)	1.12 (0.07–2.16)	1.19 (0.11–2.27)	−0.76 (− 1.74–0.22)	−0.19 (− 1.16–0.78)
2	1.42 (0.60–2.23)	1.00 (0.15–1.86)	0.46 (− 0.25–1.17)	0.83 (0.11–1.55)	3.77 (2.14–5.40)	2.73 (1.01–4.45)	0.54 (− 1.14–2.21)	1.48 (− 0.21–3.16)	0.50 (− 0.69–1.69)	0.69 (−0.56–1.94)	−0.75 (− 2.01–0.51)	0.12 (− 1.13–1.37)
3	1.71 (0.65–2.78)	1.27 (0.15–2.38)	−0.18 (− 1.41–1.05)	0.40 (− 0.84–1.64)	4.61 (2.48–6.74)	3.27 (1.03–5.50)	− 0.34 (− 3.24–2.54)	1.12 (− 1.77–4.02)	− 0.25 (− 1.81–1.32)	0.22 (− 1.42–1.86)	−2.43 (− 4.59- -0.26)	−1.03 (− 3.16–1.11)
≥4	2.49 (1.34–3.64)	2.04 (0.84–3.24)	1.30 (− 0.55–3.16)	1.88 (0.03–3.73)	6.09 (3.80–8.39)	4.83 (2.43–7.24)	2.03 (− 2.32–6.38)	3.41 (− 0.91–7.73)	1.00 (− 0.66–2.67)	1.47 (− 0.27–3.21)	− 1.42 (− 4.72–1.87)	− 0.03 (− 3.25–3.18)
*p* value	< 0.001	0.008	0.197	0.020	< 0.001	0.000	0.673	0.147	0.201	0.187	0.144	0.891
*p* linear trend	< 0.001	< 0.001	0.060	0.001	< 0.001	< 0.001	0.275	0.025	0.320	0.266	0.022	0.681
At least one livebirth												
No	Ref.	Ref.	Ref.	Ref.	Ref.	Ref.	Ref.	Ref.	Ref.	Ref.	Ref.	Ref.
Yes	1.39 (0.78–2.00)	0.96 (0.30–1.62)	0.49 (0.01–0.97)	0.79 (0.29–1.29)	3.60 (2.38–4.82)	2.50 (1.18–3.82)	0.67 (−0.46–1.80)	1.43 (0.27–2.59)	0.74 (− 0.14–1.63)	0.97 (0.01–1.93)	−0.91 (−1.76--0.07)	−0.16 (− 1.02–0.69)
*p* value	< 0.001	0.004	0.047	0.002	< 0.001	< 0.001	0.246	0.015	0.101	0.047	0.034	0.712

^a^Adjusted to maternal schooling, family income, skin color, occupational status, alcohol, smoking, physical activity, and consumption of processed and ultraprocessed foods

Additional file 1 Table S1 shows that breastfeeding did not modify the association between parity and body composition

## Discussion

In a southern Brazilian population that has been prospectively followed since birth, we found no evidence of causal association between parity and body composition at a mean age of 30 years. Parity was positively associated with body mass index and waist circumference among women. But the regression coefficients were similar among men. Suggesting, therefore, that this association must not be causal, and residual confounding is a possible explanation for the observed associations.

As previously mentioned, it has been suggested that biological changes due to pregnancy would lead to a later unbalance on body composition [3]. But, our analysis indicates that this association is not causal. In order to overcome the challenge of determining causation, the assumptions of exchangeability (no confounding), positivity (all covariate strata has exposed and unexposed subjects), and consistency (exposure must be defined with enough specificity that different variants of exposure do not have different effects on the outcome) are essential to translate evidence from observational settings [15].

Controlling for confounding variables may remove bias, but they must be perfectly measured and all non-causal pathways should be closed [6]. Effect estimates reported by observational studies can be distorted by confounding. Measurement error in a confounder and unmeasured confounders generally results in incomplete adjustment and the association of interest may be biased in any direction [16]. The assessments of social and behavioral characteristics are two of the greatest challenges. Social and behavioral variables involve complex causal chains, different dimensions, non-specific pathways and weak causal forces [17]. Also, contextual effects may interact with these individuals’ characteristics altering the occurrence of disease [17]. In spite of adjusting our estimates to traditional social and behavioral indicators, we believe that residual confounding by these characteristics could be biasing our estimates.

In the present study, we performed a comparison to assess the likelihood of residual confounding [6] by replicating our models with the cohort men. Similar results suggest that the association of parity with body composition is not due to biological changes of pregnancy. Another study based in a British birth cohort, which used a similar strategy to assess causality, found no consistent evidence of causal association, but higher regression coefficients to women than men [6]. This fact supports the suggestion that there is no causal effect of parity on body composition and reinforces the relevance of carrying out comparisons to increase causal inference of observational studies.

The strengths of our study include the large population-based cohort followed prospectively and with a high response rate [11]. Parity was reported in the same way for women and men. Furthermore, the large number of socioeconomic and lifestyle characteristics assessed in a standardized way reduces the likelihood of residual confounding. On the other hand, individuals excluded from the analyzes because of missing information on the outcomes were more likely to have lower socioeconomic status and an unhealthy lifestyle. This difference may have introduced selection bias to our results if the losses were related to the exposure as well as to the outcomes. The analyses replication with cohort’s men allow us to discuss the causal effect of parity on body composition measures of women.

## Conclusions

In conclusion, our study suggests that there is no causal effect of parity on body composition at age 30 years. Further studies should focus on elucidating the social and behavioral characteristics which may be biasing the association between parity and body composition.

## Additional files

Additional file 1: Table S1. Adjusted regression coefficients of the association of parity and body composition outcomes, according to breastfeeding time. We categorized the mean breastfeeding time in four categories and built regressions models of the association of parity and body composition outcomes adjusted for the covariates maternal schooling, family income, skin color, occupational status, alcohol, smoking, physical activity, and consumption of processed and ultraprocessed foods. (PDF 56 kb)

## Abbreviations

BMI: Body mass index
DXA: Dual-energy x-ray absorptiometry
IPAQ: International physical activity questionnaire
