# Supplementary material for: Is there a causal effect of parity on body composition: a birth cohort study
Source: BMC Public Health. 2018 Feb 22;18:215. doi: 10.1186/s12889-018-5089-2 (PMC5822479; doi:10.1186/s12889-018-5089-2)
Supplement: Supplementary file 1 — Adjusted regression coefficients of the association of parity and body composition outcomes, according to breastfeeding time. We categorized the mean breastfeeding time in four categories and built regressions models of the association of parity and body composition outcomes adjusted for the covariates maternal schooling, family income, skin color, occupational status, alcohol, smoking, physical activity, and consumption of processed and ultraprocessed foods. (PDF 56 kb) [file 12889_2018_5089_MOESM1_ESM.pdf]

Supplementary Table 1 – Adjusted [a] regression coefficients of the association of parity and body composition outcomes, according to breastfeeding time [b].

Parity		Body mass index (kg/m^2)	Waist circumference (cm)	Fat mass (%)
**Breastfed <1 month**				
	1	Ref.	Ref.	Ref.
	2	0.00 (-1.44 – 1.44)	0.16 (-2.70 – 3.03)	-1.16 (-3.26 – 0.93)
	3	0.86 (-1.06 – 2.78)	1.33 (-2.50 – 5.15)	-0.40 (-3.22 – 2.41)
	>3	1.61 (-0.56 – 3.77)	2.09 (-2.22 – 6.41)	0.89 (-2.28 – 4.05)
p value		0.433	0.752	0.585
**Breastfed 1 |--| 3 months**				
	1	Ref.	Ref.	Ref.
	2	1.91 (-0.04 – 3.87)	3.88 (0.08 – 7.68)	1.79 (-0.85 – 4.44)
	3	1.95 (-0.27 – 4.17)	3.86 (-0.44 – 8.17)	-0.92 (-4.00 – 2.15)
	>3	2.59 (0.17 – 5.01)	5.23 (0.54 – 9.92)	1.47 (-1.78 – 4.71)
p value		0.118	0.097	0.252
**Breastfed 3 --| 6 months**				
	1	Ref.	Ref.	Ref.
	2	-1.17 (-2.72 – 0.37)	-1.68 (-4.94 – 1.59)	-1.39 (-3.86 – 1.08)
	3	-1.80 (-3.92 – 0.32)	-1.25 (-5.71 – 3.20)	-2.01 (-5.34 – 1.32)
	>3	1.36 (-1.48 – 4.19)	4.75 (-1.23 – 10.73)	2.57 (-1.90 – 7.05)
p value		0.106	0.187	0.212
**Breastfed >6 months**				
	1	Ref.	Ref.	Ref.
	2	2.86 (0.13 – 5.58)	5.69 (0.17 – 11.21)	0.18 (-3.64 – 4.01)
	3	3.54 (-2.53 – 9.61)	9.20 (-3.09 – 21.49)	3.43 (-4.83 – 11.68)
	>3	1.14 (-1.75 – 4.03)	3.43 (-2.42 – 9.28)	-1.86 (-5.81 – 2.08)
p value		0.161	0.113	0.614
**Interaction test**		0.342	0.255	0.851

a Adjusted to maternal schooling, family income, skin color, occupational status, alcohol, smoking, physical activity, and consumption of processed and ultraprocessed foods.
b Mean breastfeeding time per child
